# A Multicenter Study on Unnecessary Rebiopsies in CT‐Guided Percutaneous Transthoracic Needle Biopsy of Pulmonary Lesions

**DOI:** 10.1002/cam4.71228

**Published:** 2025-09-29

**Authors:** Yangfan He, Huanhuan He, Yubo Cai, Shanshan Lyu, Zhengyi Zhou, Chengyou Zheng, Xinke Zhang, Jinling Duan, Jierong Chen, Jiewei Chen

**Affiliations:** ^1^ State Key Laboratory of Oncology in South China, Guangdong Provincial Clinical Research Center for Cancer Sun Yat‐Sen University Cancer Center Guangzhou China; ^2^ Department of Pathology Sun Yat‐Sen University Cancer Center Guangzhou China; ^3^ Department of Pathology Jiangmen Central Hospital Jiangmen China; ^4^ Department of Pathology Guangdong Provincial People's Hospital Guangzhou China

**Keywords:** CT‐guided percutaneous transthoracic needle biopsy of pulmonary, lung cancer, multidisciplinary team, pathological diagnosis, rebiopsy

## Abstract

**Background and Objective:**

Computed tomography‐guided percutaneous transthoracic needle biopsy of pulmonary lesions (PTNBP) is widely used for diagnosing and managing lung cancer. However, rebiopsies are often required due to insufficient specimens for definitive pathological diagnosis and molecular testing, which increase patient burden and reduce treatment efficiency. This study aims to investigate the characteristics of rebiopsies and propose strategies to minimize unnecessary rebiopsies.

**Methods:**

A total of 12,882 PTNBP cases from three centers were analyzed from January 2018 to December 2022. Attribution analysis was conducted for the rebiopsy cases, and descriptive analysis was conducted for clinical parameters.

**Results:**

The rebiopsy rate was 6.5% (841/12,882), with 31.0% (261/841) of rebiopsies due to insufficient specimens. Among these cases, 87.0% (227/261) were stage III–IV, 89.7% (234/261) had poorly differentiated tumors, and 80.1% (209/261) were non‐small cell lung cancer (NSCLC). The proportion of molecular testing in stage IV cases was significantly higher than in stage I–III cases (44.8% vs. 25.3%, *p* = 0.0022). Poorly differentiated cases required significantly more biomarkers in immunohistochemistry and special staining than moderately and well‐differentiated cases (8.1 vs. 4.0, *p* = 0.0039). Insufficient tissue rates were significantly higher in cases with only one biopsy core compared to those with more cores (2.4% vs. 1.5%, *p* = 0.0088).

**Conclusion:**

Patients with advanced‐stage disease, poorly differentiated tumors, and NSCLC undergoing PTNBP are more likely to require rebiopsy due to insufficient specimens. Increasing the number of biopsy cores (≥ 2) and implementing one‐stop management for pathological assessment may effectively reduce unnecessary rebiopsies in PTNBP.

**Abbreviations**
CT: computed tomography
GDPPH: Guangdong Provincial People's Hospital
H&E: hematoxylin–eosin
IHC: immunohistochemistry
JMCH: Jiangmen Central Hospital
MDT: multidisciplinary team
NSCLC: non‐small cell lung cancer
PTNBP: percutaneous transthoracic needle biopsy of pulmonary
SYSUCC: Sun Yat‐sen University Cancer Center

## 1 Introduction

Lung cancer is the second most common cancer and the leading cause of cancer‐related deaths worldwide [1]. Computed tomography (CT)‐guided percutaneous transthoracic needle biopsy of pulmonary lesions (PTNBP) has been widely utilized for the diagnosis and treatment of lung cancer [2, 3]. However, it carries risks of complications such as pneumothorax, hemoptysis, air embolism, and hemothorax [4]. In clinical practice, it is common for the same patient to undergo multiple biopsies—referred to as rebiopsies—due to various factors such as cancer progression, acquired drug resistance, participation in clinical trials, insufficient initial biopsy material, and others [5, 6]. While some rebiopsies are reasonable [7, 8, 9], others, such as those due to insufficient specimens for definitive pathological diagnosis and molecular testing, are avoidable. These unnecessary rebiopsies significantly increase the burden on the patient and reduce the efficiency of medical treatment. Despite this, unnecessary rebiopsies have not received enough attention in clinical practice, and research on this issue is scarce.

There are several limitations in the pathological diagnosis of PTNBP specimens, mainly concerning the sufficiency of tissue, the typicality of histological morphology, and the heterogeneity of lung cancer histology and biology [10, 11]. In clinical practice, CT‐guided lung biopsy often yields limited tissue samples, resulting in minimal diagnostic material. The pathological diagnosis of poorly differentiated tumors is particularly challenging [12]. In advanced cases of lung cancer, it is essential not only to ensure a definitive pathological diagnosis and classification but also to obtain sufficient tissue for molecular examinations related to precision treatment, highlighting the value of small biopsy samples [13]. However, there is still a lack of clinical guidelines to manage these specimens efficiently, to minimize unnecessary waste and to maximize the number of tests that can be performed with a limited amount of tissue during pathological investigations.

This study retrospectively analyzed the clinical data of CT‐guided PTNBP patients across multiple centers, identifying rebiopsy cases and summarizing the reasons for rebiopsy. We investigated the clinicopathological features of cases with insufficient specimens from multiple perspectives and proposed strategies to reduce unnecessary rebiopsies. Our findings offer valuable insights for improving clinicopathological management, reducing the rebiopsy rate, and enhancing the accuracy of pathological diagnosis in PTNBP cases, thereby better supporting the needs of clinical precision treatment.

## 2 Materials and Methods

### 2.1 Participants

**FIGURE 1:**
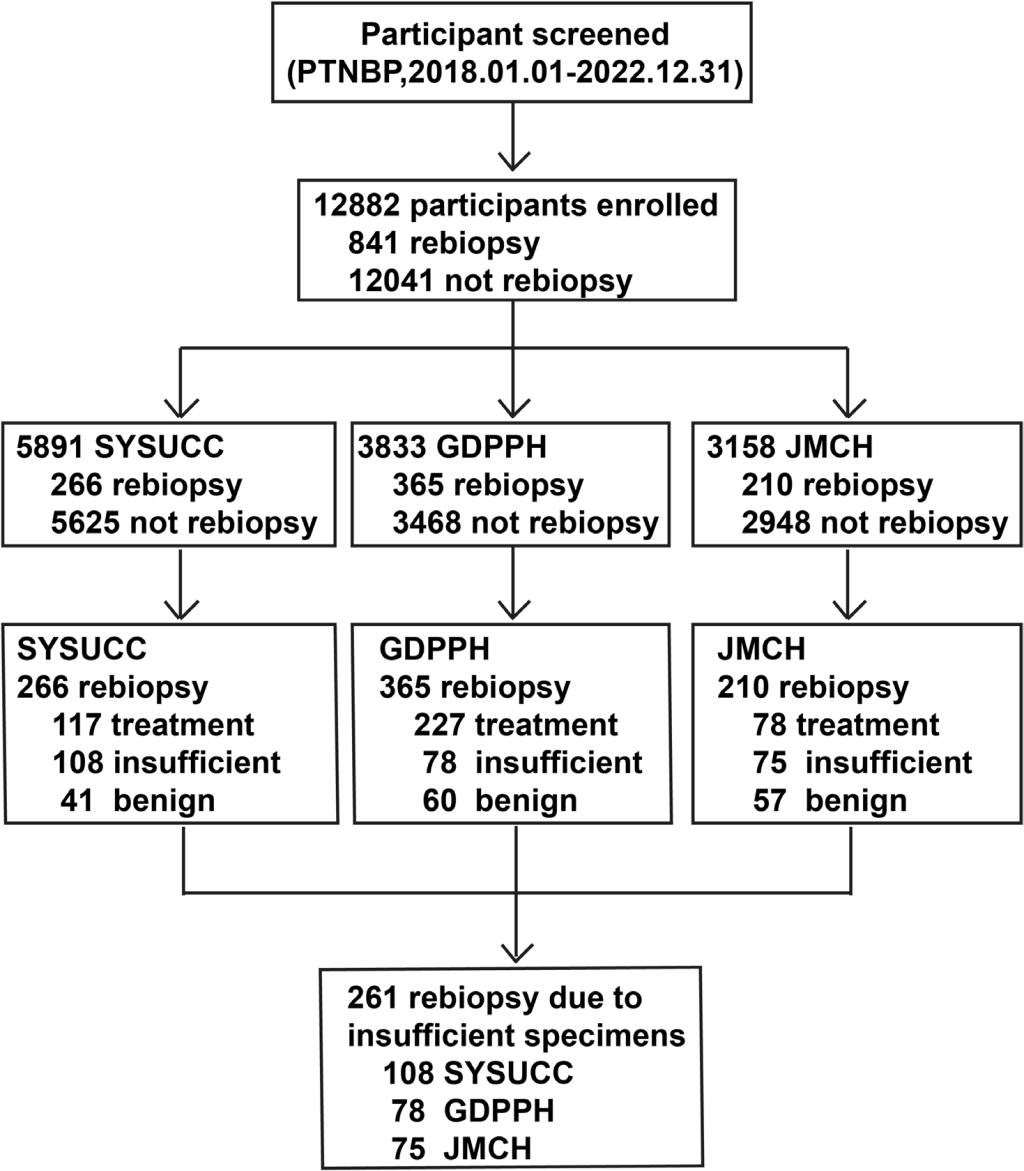
Participant enrollment procedures.

A total of 12,882 patients who underwent CT‐guided PTNBP at three institutions—Sun Yat‐sen University Cancer Center (SYSUCC), Guangdong Provincial People's Hospital (GDPPH), and Jiangmen Central Hospital (JMCH)—were included in this study, with a follow‐up period from January 2018 to December 2022 (Figure 1). In this study, rebiopsy cases are defined as patients who underwent two or more CT‐guided PTNBP procedures during the follow‐up period.

### 2.2 Data Collection

We collected clinical and pathological data from PTNBP patients, including gender, age, lesion size, biopsy date, histology diagnosis, degree of differentiation, TNM stage, lobar location, reason for rebiopsy, hematoxylin–eosin (H&E) sections, immunohistochemistry (IHC) and special staining biomarkers, molecular testing results, and other relevant information.

### 2.3 Pathological Diagnosis and Slide Scanning

In this study, the H&E‐stained slides of rebiopsy cases were retrospectively and the PTNBP cases prospectively reviewed by two clinical pathologists independently. H&E‐stained slides were digitized using whole‐slide imaging with a digital pathology scanner (Leica Aperio AT2).

### 2.4 Biopsy Technique

At each participating center, biopsy procedures were primarily conducted by specialized thoracic radiologists. All biopsies were performed using an 18‐Gauge core needle within a coaxial cutting needle system (BARD MC1816, 18G × 16 cm; BioPince MCXS1815BP, 18G × 15 cm).

Conventional spiral CT scanning was utilized to identify and localize the lesion. The needle insertion point and trajectory were determined using the CT laser positioning system. Following routine disinfection and application of sterile drapes, local infiltration anesthesia was administered with 2% lidocaine. Under continuous guidance by spiral CT scanning, the needle was accurately inserted through the skin into the tumor. The biopsy specimen was immediately placed in formalin fixative and sent for pathological evaluation.

### 2.5 Statistical Analysis

GraphPad Prism 8 was employed for graph generation, while SPSS 16.0 (SPSS Inc., Chicago, IL, USA) was used for statistical analysis. An unpaired *t*‐test was applied to compare the number of IHC and special staining biomarkers between clinicopathological parameters and pathological detection needs, and the number of biopsy cores between rebiopsy cases with insufficient specimens at the first biopsy and cases without rebiopsy. The Chi‐square test was conducted to compare differences in molecular detection proportions among different clinicopathological parameters and the insufficiency rate of different biopsy cores. A *p* value of less than 0.05 (two‐tailed) was considered statistically significant.

## 3 Results

### 3.1 Participants

A total of 12,882 patients who underwent CT‐guided PTNBP at three centers (SYSUCC, GDPPH, and JMCH) were retrospectively analyzed in this study. Among them, 841 patients underwent rebiopsy from January 2018 to December 2022, and the overall rebiopsy rate was 6.5% (841/12,882) (Figure S1). Of these rebiopsy cases, 261 were due to insufficient initial specimens for a definitive pathological diagnosis or molecular analysis (Figure 1).

### 3.2 Reason for Rebiopsy

Reasons for rebiopsy were categorized into three groups: treatment‐related factors, insufficient specimens, and benign lesions, based on a retrospective review of medical records, clinical guidelines, and expert consensus. Among the 841 patients who underwent rebiopsy, 422 (50.2%) underwent rebiopsy due to treatment‐related indications, such as disease progression, acquired drug resistance, or participation in clinical trials. A total of 261 patients (31.0%) required rebiopsy because the initial specimens were inadequate for clinicopathological diagnosis and molecular testing. Furthermore, 158 patients (18.8%) underwent rebiopsies when the initial biopsy indicated benign lesions, such as inflammatory changes, while imaging findings and clinical symptoms suggested potential malignancy (Figure S2).

**FIGURE 2:**
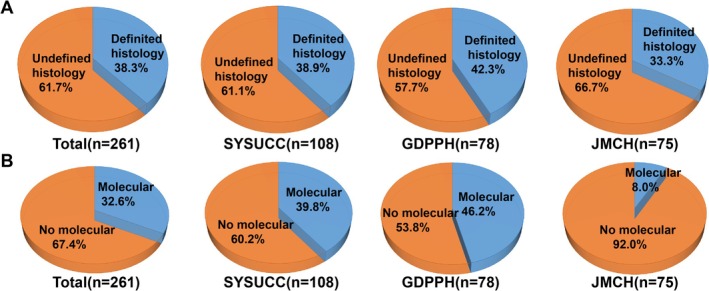
Reason for rebiopsy due to insufficient specimens. (A) Proportion of cases with an undefined pathological diagnosis at the initial biopsy due to insufficient specimens. (B) Proportion of cases requiring molecular detection using rebiopsy tissue due to insufficient specimens.

Among the 261 cases with insufficient specimens, 161 (61.7%) did not receive a definitive pathological classification from the initial biopsy and required rebiopsy to confirm the pathological subtype. Additionally, 85 (32.6%) patients underwent molecular detection with rebiopsy tissue because of insufficient initial specimens (Figure 2).

### 3.3 Clinicopathological Characteristics of Rebiopsy Patients due to Insufficient Initial Specimens

Among the 261 cases with insufficient specimens, 177 (67.8%) were male and 84 (32.2%) were female, with a median age of 63 years and a median tumor size of 4.2 cm (Table 1, Figure S3B). The median interval between the first and second biopsies was 0.2 months (Table 1, Figure S3A). Of these patients, 209 (80.1%) were diagnosed with non‐small cell lung cancer (NSCLC), while 29 (11.1%) had challenging diagnoses, including atypical carcinomas, lymphomas, and sarcomas (Table 1, Figure S3C). Poorly differentiated tumors constituted 89.7% of all cases, with well‐to‐moderately differentiated tumors making up the remaining 10.3% (Table 1, Figure S3D). According to TNM staging criteria, 13% of patients were classified as stage I–II, 20.3% as stage III, and 66.7% as stage IV (Table 1, Figure S3E). Regarding the biopsy sites, 158 (60.6%) were in the upper lobe, and 153 (58.6%) were in the right lobe (Table 1, Figure S3F).

**TABLE 1:** Clinicopathological parameters of rebiopsy patients with insufficient specimens.

Variable	All groups	SYSUCC	GDPPH	JMCH
	(*n* = 261)	(*n* = 108)	(*n* = 78)	(*n* = 75)
Gender				
Male	177 (67.8%)	75 (69.4%)	53 (67.9%)	49 (65.3%)
Female	84 (32.2%)	33 (30.6%)	25 (32.1%)	26 (34.7%)
Age (years)^a^	63.0	61.5	61.0	67.0
Tumor size (cm)^a^	4.2	3.9	5.2	3.8
Rebiopsy interval time (month)^a^	0.2	0.5	0.2	0.2
Histology (final)				
Non‐small cell lung cancer	209 (80.1%)	74 (68.5%)	69 (88.5%)	66 (88.0%)
Small cell lung cancer	8 (3.1%)	6 (5.6%)	1 (1.3%)	1 (1.3%)
Metastasis lung cancer	15 (5.7%)	11 (10.2%)	2 (2.6%)	2 (2.7%)
Difficult diagnostic cancer	29 (11.1%)	17 (15.7%)	6 (7.7%)	6 (8.0%)
Differentiation				
Well‐moderate	27 (10.3%)	19 (17.6%)	3 (3.8%)	5 (6.7%)
Poor‐undifferentiated	234 (89.7%)	89 (82.4%)	75 (96.2%)	70 (93.3%)
TNM stage				
I–II	34 (13.0%)	13 (12.0%)	5 (6.4%)	16 (21.3%)
III	53 (20.3%)	17 (15.7%)	19 (24.4%)	17 (22.7%)
IV	174 (66.7%)	78 (72.2%)	54 (69.2%)	42 (56.0%)
Lobar site				
Upper‐left	72 (27.6%)	40 (37.0%)	16 (20.5%)	16 (21.3%)
Lower‐left	36 (13.8%)	13 (12.0%)	12 (15.4%)	11 (14.7%)
Upper‐right	86 (33.0%)	30 (27.8%)	32 (41.0%)	24 (32.0%)
Right‐middle	11 (4.2%)	4 (3.7%)	3 (3.9%)	4 (5.3%)
Lower‐right	56 (21.5%)	21 (19.4%)	15 (19.2%)	20 (26.7%)

^a^ Data are the median.

Cases that underwent rebiopsy due to inadequate tissue samples showed several key differences compared to those rebiopsied for treatment‐related reasons. These cases involved older patients (61.8 vs. 59.1, *p* = 0.0014), had a shorter interval between initial biopsy and rebiopsy (1.2 vs. 15.1, *p* < 0.0001), included more males (67.8% vs. 54.5%, *p* = 0.0006), and showed a higher rate of uncommon or other pathological types (19.9% vs. 12.1%, *p* = 0.0054) (Table S1).

### 3.4 Pathological Detection Needs of Rebiopsy Patients With Insufficient Specimens

To further characterize cases with insufficient tissue, we analyzed their pathological detection needs from the aspects of H&E staining, IHC (TTF1, p40, Napsin A, CK5/6, p63, Ki‐67, etc.), and molecular detection (ALK, EGFR, ROS1, etc.). The results revealed that six sections were stained with H&E at each of the three participating centers (Figure S4A, Table 2). The average number of biomarkers for IHC and special staining in the first, second, and combined biopsies was 3.2, 4.4, and 7.7, respectively (Figure S4B, Table 2). The most frequently utilized IHC biomarkers were primarily TTF‐1, p40, Napsin A, CK5/6, p63, and Ki 67 (Table S2). The proportion of cases undergoing molecular detection was 13.8% in the first biopsy, 28.7% in the second biopsy, and 38.3% in both biopsies (Figure S4C, Table 2). The molecular markers with the highest detection rates are predominantly ALK, EGFR, and ROS1 (Table S2).

**TABLE 2:** Pathological detection needs of rebiopsy patients with insufficient specimens.

	H&E sections	IHC and special staining		Proportion of molecular
		Number of biomarker (Mean ± SEM)	Proportion	
1st	6	3.238 ± 0.2985	41.4% (108/261)	13.8% (36/261)
2nd	6	4.418 ± 0.3253	52.5% (137/261)	28.7% (75/261)
1st + 2nd	12	7.655 ± 0.4321	76.2% (199/261)	38.3% (100/261)

**FIGURE 3:**
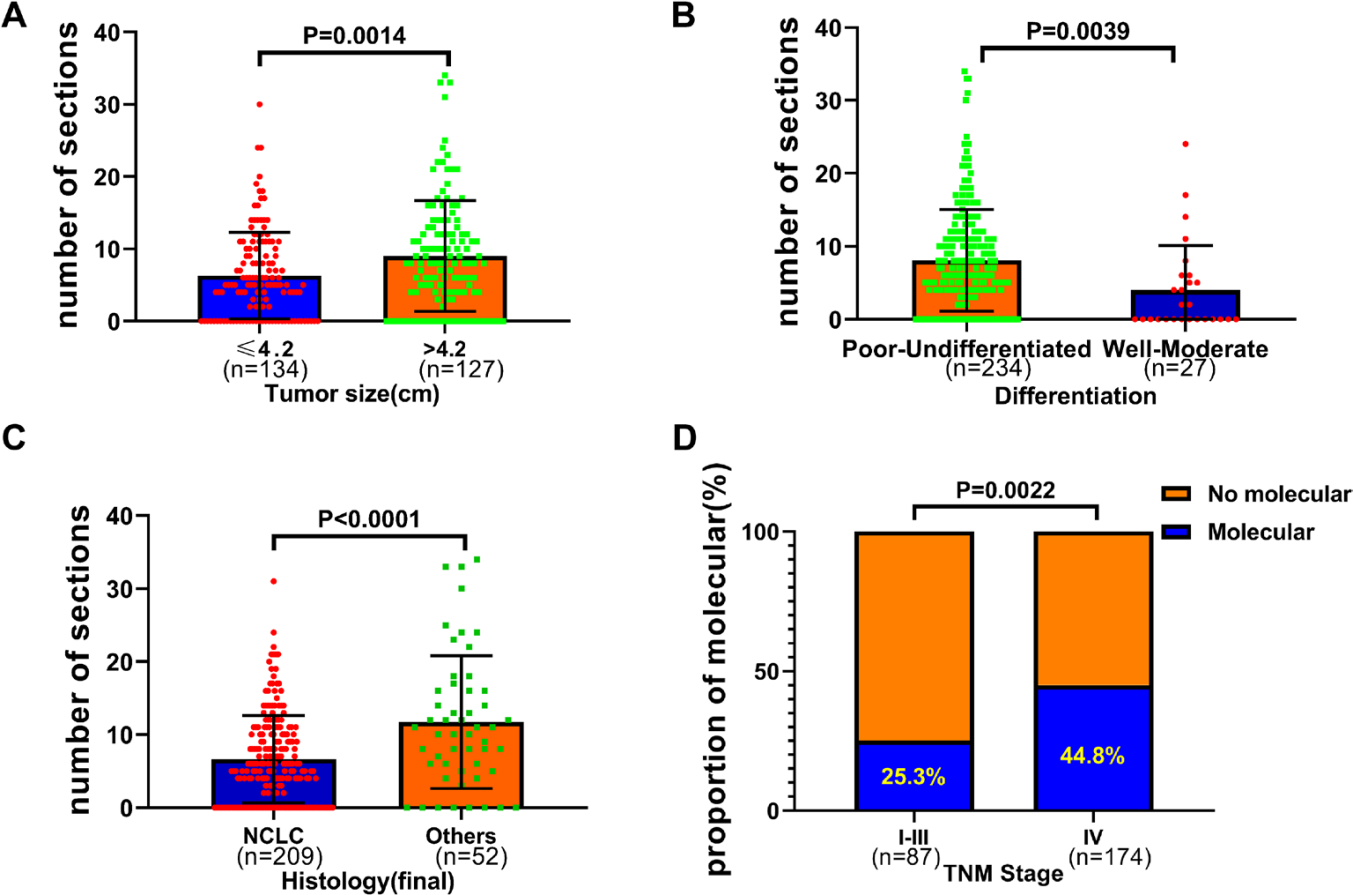
Pathological detection needs of rebiopsy patients with insufficient specimens. (A) Biomarker count for IHC and special staining in relation to different tumor sizes. (B) Biomarker count for IHC and special staining based on different degrees of differentiation. (C) Biomarker count for IHC and special staining in NSCLC versus other pathological types (including small cell lung cancer, metastatic lung cancer, and difficult‐to‐diagnose cancers). (D) Proportion of molecular detection with different TNM stages.

We also analyzed the relationship between clinicopathological parameters and pathological detection needs. The results showed that the number of biomarkers for IHC and special staining in cases with larger tumors (> 4.2 cm) was significantly higher than in cases with smaller tumors (≤ 4.2 cm) (9.1 vs. 6.3, respectively, *p* = 0.0014; Figure 3A, Tables S3 and S4). The number of biomarkers for IHC and special staining in poorly differentiated cases was significantly higher than in well‐to‐moderately differentiated cases (8.1 vs. 4.0, respectively, *p* = 0.0039; Figure 3B, Table S3). The biomarker number of IHC and special staining in NSCLC was significantly lower than that in other pathological subtypes (6.6 vs. 11.7, respectively, *p* < 0.0001; Figure 3C, Tables S3 and S4). The proportion of molecular detection in stage IV patients was significantly higher than in stage I–III cases (25.3% vs. 44.8%, respectively, *p* = 0.0022; Figure 3D, Table S3).

### 3.5 Correlation Analysis Between Insufficient Tissue Rate and Number of Biopsy Cores

**FIGURE 4:**
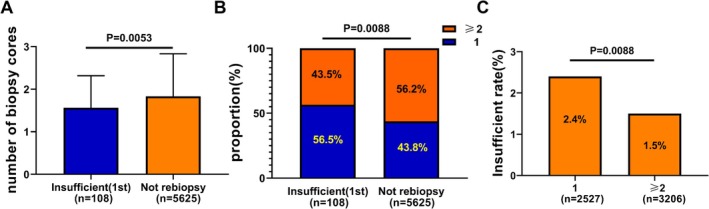
Correlation analysis between rebiopsy rate and number of biopsy cores. (A) Analysis of the difference in the number of biopsy cores (Unpaired *t*‐test). (B) Proportion of different biopsy cores (Chi‐square test). (C) Insufficient tissue rate associated with different biopsy cores (Chi‐square test).

To further investigate the characteristics of cases with insufficient tissue, we compared the number of biopsy cores from the first biopsy between patients with insufficient tissue and those who did not require rebiopsy. The results indicated that the average number of biopsy cores in non‐rebiopsy cases was significantly higher than in rebiopsy cases (1.8 vs. 1.6, respectively, *p* = 0.0053; Figure 4A). Additionally, the proportion of cases with two or more biopsy cores was significantly higher in non‐rebiopsy cases compared to those with insufficient tissue (56.2% vs. 43.5%, respectively, *p* = 0.0088; Figure 4B). The rate of insufficient tissue for cases with only one biopsy core was significantly higher than for cases with two or more cores (2.4% vs. 1.5%, respectively, *p* = 0.0088; Figure 4C).

### 3.6 Clinical Practice Recommendations to Reduce Unnecessary Rebiopsy due to Insufficient Specimens

To identify solutions for reducing the rebiopsy rate, we retrospectively reviewed the pathology images of 266 rebiopsy patients (532 biopsies) from SYSUCC (Table S5). We found no significant morphological differences among the six H&E‐stained sections in each biopsy case. Morphological differences were defined as changes observed under the microscope, such as from no malignant cells to malignant cells or vice versa, or from large areas of malignant cells to small focal areas or vice versa. To eliminate factors such as fading and sampling errors, we prospectively observed 547 cases that underwent PTNBP (Table S5). The results showed no significant differences in histomorphology under continuous H&E sections for either typical lung cancer types (such as adenocarcinoma, Figure S5A) or atypical types (such as poorly differentiated carcinoma, Figure S5B). This suggests that staining H&E on continuous sections for PTNBP tissues is unnecessary because it does not significantly reduce diagnostic accuracy or the rate of missed diagnosis.

**FIGURE 5:**
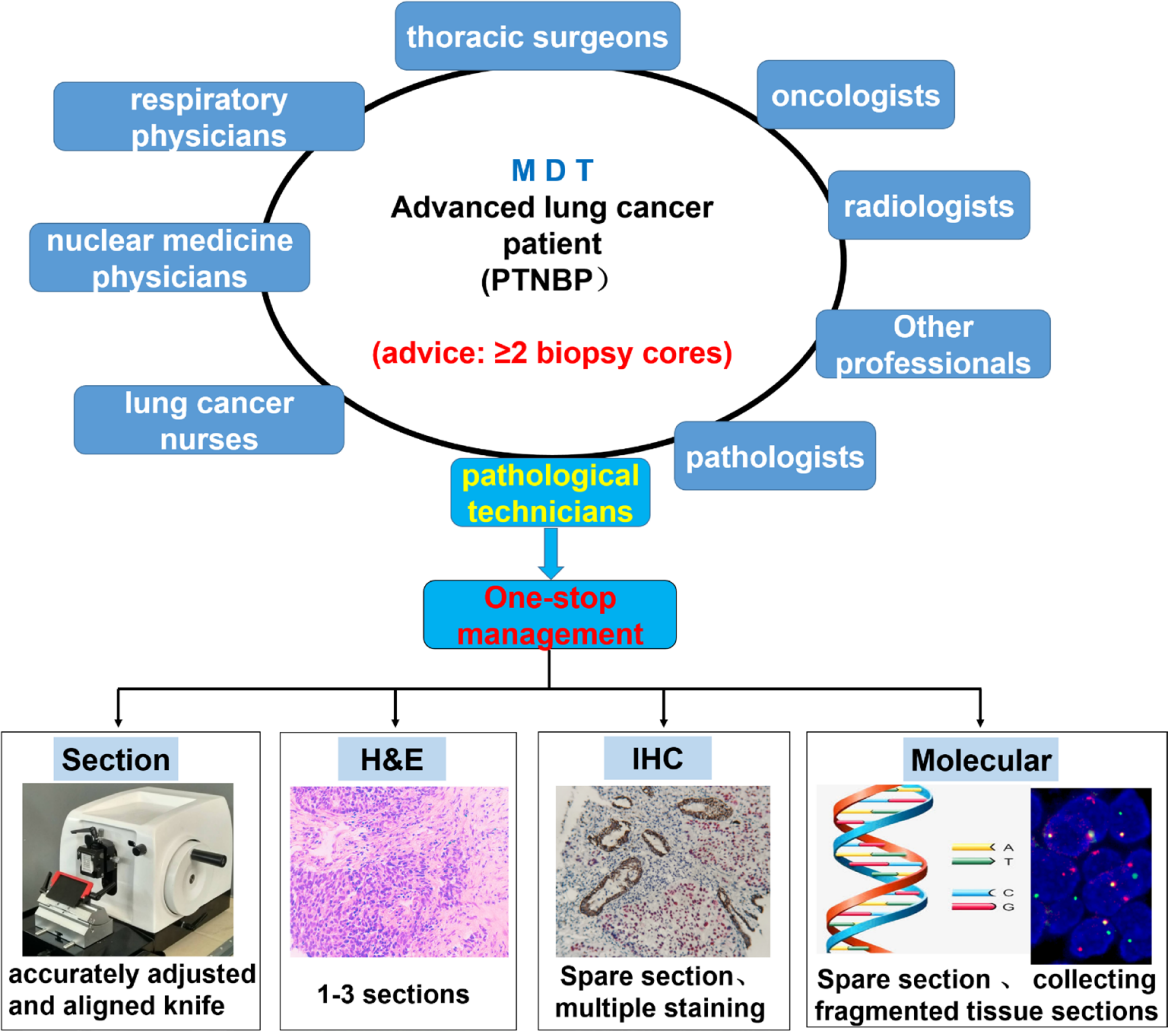
Clinical practice recommendations to reduce unnecessary rebiopsy due to insufficient specimens.

First, clinicians should fully communicate with patients suspected of advanced lung cancer, advising them to obtain two or more biopsy cores to reduce the risk of rebiopsy. Second, for lung cancer patients requiring biopsy for pathological diagnosis and molecular detection, it is recommended that pathology technicians manage the entire process of PTNBP specimen handling, including one‐stop management and operation, after receiving samples. During the sectioning process, the knife should be adjusted and aligned as accurately as possible. Multiple continuous sections should be prepared simultaneously, with 1–3 sections used for H&E staining and the others reserved for IHC and molecular detection. Fragmented tissue sections generated during rough sectioning can be collected and utilized for molecular detection. Moreover, multiple staining techniques for IHC can be considered to improve the utilization rate of sections in institutions with appropriate experimental conditions (Figure 5).

## 4 Discussion

With the widespread use of CT‐guided PTNBP in diagnosing and treating lung cancer, an increasing number of patients are undergoing multiple PTNBP procedures (rebiopsies) in clinical practice. This study retrospectively analyzed 12,882 cases of CT‐guided PTNBP across three centers, identifying a total of 841 rebiopsy cases, yielding a rebiopsy rate of 6.5%.

Currently, the distribution of rebiopsy cases for different reasons has not been reported. This study summarized the distribution of rebiopsy cases based on various reasons. We found that the majority of rebiopsies (50.2%) were performed due to treatment‐related factors. Existing literature supports the necessity of rebiopsy in these cases to monitor disease progression, screen for drug resistance genes, interpret resistance mechanisms, predict therapeutic targets, and detect new tumor characteristics, thereby providing a basis for subsequent treatment decisions [6, 7, 9, 14, 15, 16, 17].

Other cases (31.0%) involved rebiopsy due to insufficient specimens from the initial biopsy, inadequate for clinicopathological diagnosis or molecular detection. The reasons for insufficient tissue spanned multiple clinical and pathological processes. Providing sufficient tissue initially or optimizing tissue utilization throughout the process might help in these patients avoid rebiopsy.

The analysis of clinicopathological characteristics of rebiopsy patients with insufficient specimens revealed that the interval between the first and second biopsy was significantly shorter compared to those undergoing rebiopsy for treatment, indicating the urgent need for pathology diagnosis and molecular subtyping. Most of the cases with insufficient tissue were poorly differentiated tumors (89.7%), which are notoriously difficult to diagnose and often require additional techniques such as IHC and special staining [12]. Notably, a significant proportion of these cases were diagnosed with NSCLC (80.1%), which is the most important pathological type of lung cancer and a primary target for molecular targeted therapy and immunotherapy [17, 18, 19]. Cases requiring rebiopsy due to insufficient tissue have a higher proportion of uncommon pathological types and often need more IHC markers for diagnosis, leading to greater tissue consumption. According to TNM staging, 87% of the cases with insufficient tissue were advanced patients (stages III–IV), and these patients required both a definitive pathological diagnosis and molecular detection to meet the demands of precision treatment [17, 18, 19]. Subsequently, we analyzed the necessity for pathological diagnosis in cases with insufficient tissue and found that approximately 60% of these cases required definite pathological classification, which was an important basis for subsequent treatment decision‐making [20]. In clinical practice, pathologists typically observe the morphology of lung biopsy specimens using H&E staining for preliminary diagnosis. When a differential diagnosis is required due to atypical histological morphology, additional techniques such as IHC and special staining are used to assist in diagnosis. Our analysis of pathology images from the study cohort revealed that six consecutive sections were used for histological preliminary diagnosis in each H&E staining across the three centers. The data showed no significant difference in histological morphology among these six consecutive sections, suggesting that it might be unnecessary to use continuous sections for H&E staining in the morphological preliminary screening of lung biopsy specimens. By reducing the number of tissues stained with H&E, more tissue samples could be preserved for subsequent IHC and molecular testing, potentially reducing the need for unnecessary rebiopsy. IHC and special staining were performed in 76.2% of patients with insufficient tissue, with an average of 7.7 biomarkers used for these procedures. Due to tissue heterogeneity and other factors, some detection items were repeated, increasing the overall medical costs.

The number of biomarkers used for IHC and special staining in cases with larger tumors (> 4.2 cm) was significantly higher than in those with smaller tumors (≤ 4.2 cm), which may be attributed to tumor heterogeneity. Similarly, the number of biomarkers used in poorly differentiated cases was significantly higher than in well‐to‐moderately differentiated cases, indicating the greater challenge in diagnosing poorly differentiated tumors. When NSCLC is poorly differentiated and cannot be further classified by morphology alone, IHC and special staining become essential for diagnosis.

The subsequent analysis of the need for molecular testing in cases with insufficient tissue revealed that at least 38.3% of these patients required molecular testing, with at least 28.7% undergoing rebiopsy specifically for this purpose. The demand for molecular testing was significantly higher among stage IV patients compared to those in stages I–III (44.8% vs. 25.3%).

Additionally, to further characterize patients with insufficient tissue, we analyzed the number of biopsy cores. The results indicated that the number of biopsy cores in non‐rebiopsy cases was significantly higher than in rebiopsy cases during the first biopsy (1.8 vs. 1.6). Furthermore, the proportion of cases with two or more biopsy cores was significantly higher in non‐biopsy cases compared to those with insufficient tissue (56.2% vs. 43.5%). The insufficiency rate for cases with only one biopsy core was significantly higher than for those with two or more cores (2.4% vs. 1.5%). These findings suggested that the relatively small amount of tissue in the first biopsy was a significant factor contributing to the need for rebiopsy. In addition, Beck et al. showed that increasing the number of biopsy specimens improved the sensitivity of pathological diagnosis for pulmonary nodules, but the improvement in diagnostic performance was not significant when the number increased from two to three [21]. Lim et al. suggested that three coaxial needle passes might be optimal in the diagnosis of lung malignancy [22]. Kim et al. found that there was no evidence of an association between the number of core biopsy samples obtained and any postprocedural complications [23]. Based on these findings, it is advisable to obtain two or more biopsy cores whenever possible for patients with advanced lung cancer requiring molecular testing. This approach could help preserve sufficient tissue for subsequent molecular testing, potentially reducing the need for unnecessary rebiopsies due to insufficient tissue and improving the success rate of initial diagnosis and subtyping [8, 24, 25].

Our study highlighted that multistation and subcenter operations in the pathological technical process of lung biopsy samples across the three centers significantly contributed to unnecessary tissue loss. In the era of precision medicine, the multidisciplinary team (MDT) model for lung cancer is increasingly utilized, as it effectively enhances the accuracy of clinical decision‐making. Currently, the MDT members for lung cancer typically include thoracic surgeons, physicians, oncologists, radiologists, pathologists, and nurses, among others. Each team member provides insights from their professional perspective [26, 27, 28, 29].

Pathologists mainly provide histopathological diagnoses and molecular test reports. However, there is limited discussion on managing and planning lung biopsy specimens, optimizing testing with minimal samples, or determining the best biopsy strategy for specific cases. Including pathology technicians in the lung cancer MDT, especially for advanced‐stage or atypical imaging cases, could be beneficial. These technicians, with their expertise in pathological technology, could suggest more scientific and accurate detection strategies during discussions on histological treatment and subsequent testing of lung biopsy samples.

Pathology technicians can also understand the clinical diagnosis and treatment needs through MDT participation. This involvement allows them to develop scientific and reasonable plans, conduct one‐stop operations, pay attention to every detail, optimize tissue processing strategies, and maximize tissue sample use throughout the pathology assessment process. For instance, fragmented tissue can be collected during initial slicing for subsequent molecular detection. Multiple unstained slides can be continuously cut for IHC staining and molecular detection to prevent tissue loss from repeated paraffin block cutting. Additionally, double staining or multiple staining can be considered for IHC in facilities with the necessary experimental conditions, further improving section utilization. These measures not only save detection time and yield more accurate results but also help avoid unnecessary rebiopsies. The findings and recommendations from this study should be widely implemented and gradually optimized in clinical practice.

## 5 Conclusion

In conclusion, this multicenter retrospective study systematically investigated and analyzed the clinicopathological data of PTNBP cases, providing a comprehensive examination of the challenges encountered in clinical and pathological practices of PTNBP from multiple perspectives. It highlights the necessity of whole‐process management and one‐stop operation of the pathological technology workstation for PTNBP cases. Our findings provide potentially valuable recommendations for improving clinicopathological management practices, reducing the rebiopsy rate, and enhancing the accuracy of pathological diagnosis in PTNBP cases, thereby supporting the requirements for clinical precision treatment.

## Author Contributions

**Yangfan He:** data curation (equal), formal analysis (equal), investigation (equal), methodology (equal), software (equal), validation (equal), visualization (equal), writing – original draft (equal). **Huanhuan He:** data curation (equal), formal analysis (equal), investigation (equal), methodology (equal), software (equal). **Yubo Cai:** data curation (equal), investigation (equal), resources (equal), validation (equal). **Shanshan Lyu:** data curation (equal), investigation (equal), resources (equal), validation (equal). **Zhengyi Zhou:** software (equal), supervision (equal), validation (equal). **Chengyou Zheng:** validation (equal), visualization (equal). **Xinke Zhang:** investigation (equal), methodology (equal). **Jinling Duan:** supervision (equal), validation (equal), visualization (equal). **Jierong Chen:** conceptualization (equal), project administration (equal), writing – review and editing (equal). **Jiewei Chen:** conceptualization (equal), funding acquisition (equal), project administration (equal), writing – review and editing (equal).

## Ethics Statement

The studies involving human participants were reviewed and approved by the Institutional Review Board of Sun Yat‐sen University Cancer Center (B2023‐680). The patients/participants provided their written informed consent to participate in this study.

## Conflicts of Interest

The authors declare no conflicts of interest.

## Supporting information


**Figure S1:** Rebiopsy rate.

**Figure S2:** Distribution of factors leading to rebiopsy.

**Figure S3:** Clinicopathological characteristics of rebiopsy patients with insufficient specimens. (A) Frequency distribution of rebiopsy interval time. (B) Distribution of tumor size. (C) Distribution of pathological histology. (D) Distribution of tumor differentiation. (E) Distribution of TNM stage. (F) Lobar site of the biopsy tissue.

**Figure S4:** Pathological detection needs of rebiopsy patients with insufficient specimens. (A) Number of sections with H&E staining. (B) Biomarker count for immunohistochemistry (IHC) and special staining. (C) Proportion of molecular detection.

**Figure S5:** Images of H&E staining with continuous sections for PTNBP tissues. (A) H&E images of lung adenocarcinoma. (B) H&E images of poorly differentiated carcinoma (10×).

**Table S1:** Differences in clinicopathological parameters between rebiopsy cases with insufficient specimens and those due to treatment.

**Table S2:** The biomarker of IHC and molecular testing.

**Table S3:** Relationship between clinicopathological parameters and pathological detection needs.

**Table S4:** Multivariate analyses of clinicopathological parameters for pathological detection needs (*n* = 261).

**Table S5:** Clinicopathological parameters of patients stained H&E with continuous sections.

## Data Availability

The authenticity of this article has been validated by uploading the key raw data onto the Research Data Deposit public platform (www.researchdata.org.cn).
