# Supplementary figures and images for: A Multicenter Study on Unnecessary Rebiopsies in CT‐Guided Percutaneous Transthoracic Needle Biopsy of Pulmonary Lesions
Source: Cancer Med. 2025 Sep 29;14(19):e71228. doi: 10.1002/cam4.71228 (PMC12477545; doi:10.1002/cam4.71228)

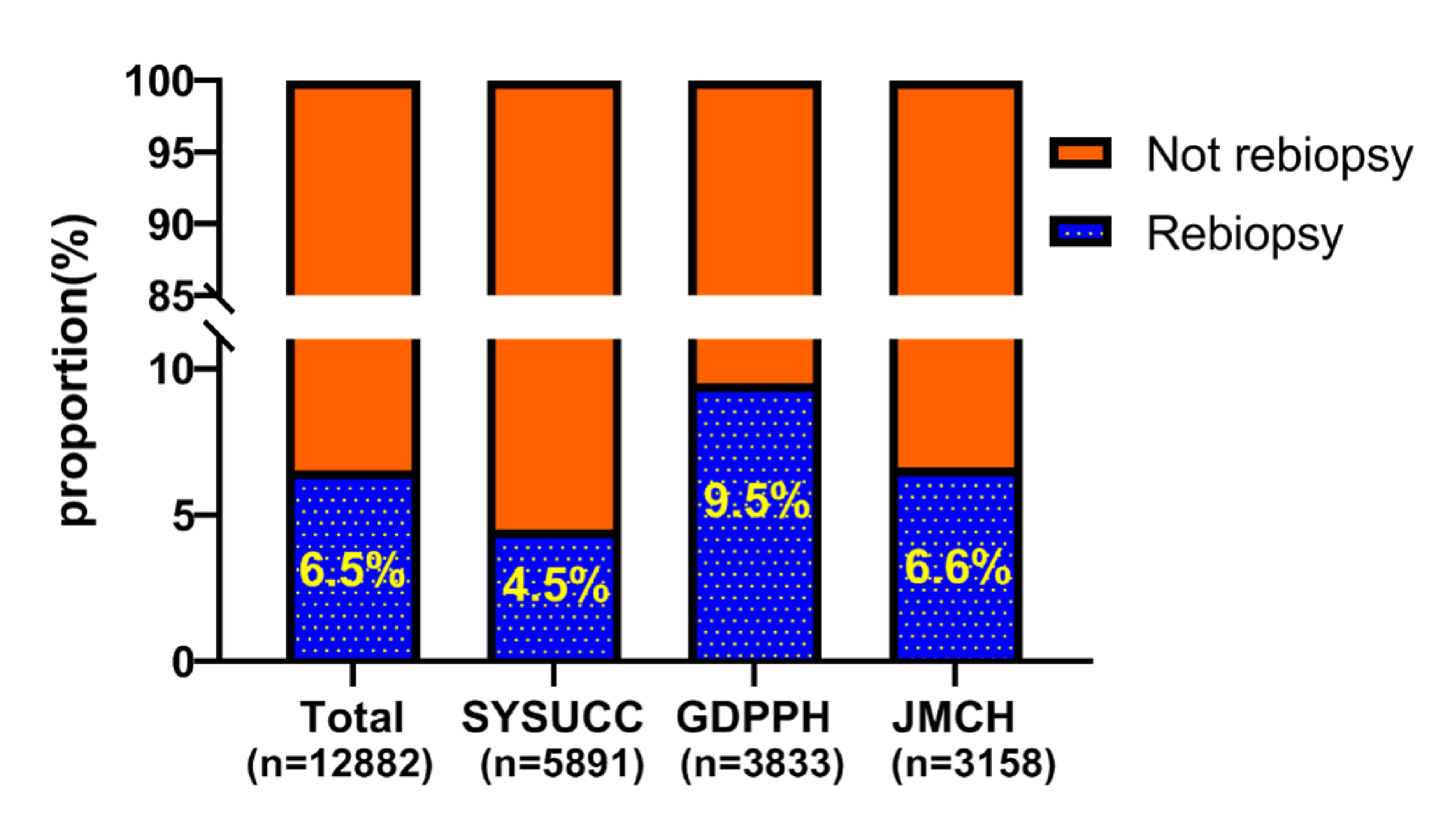

Supplement: Supplementary file 1 — Figure S1: Rebiopsy rate. [file CAM4-14-e71228-s010.tif]

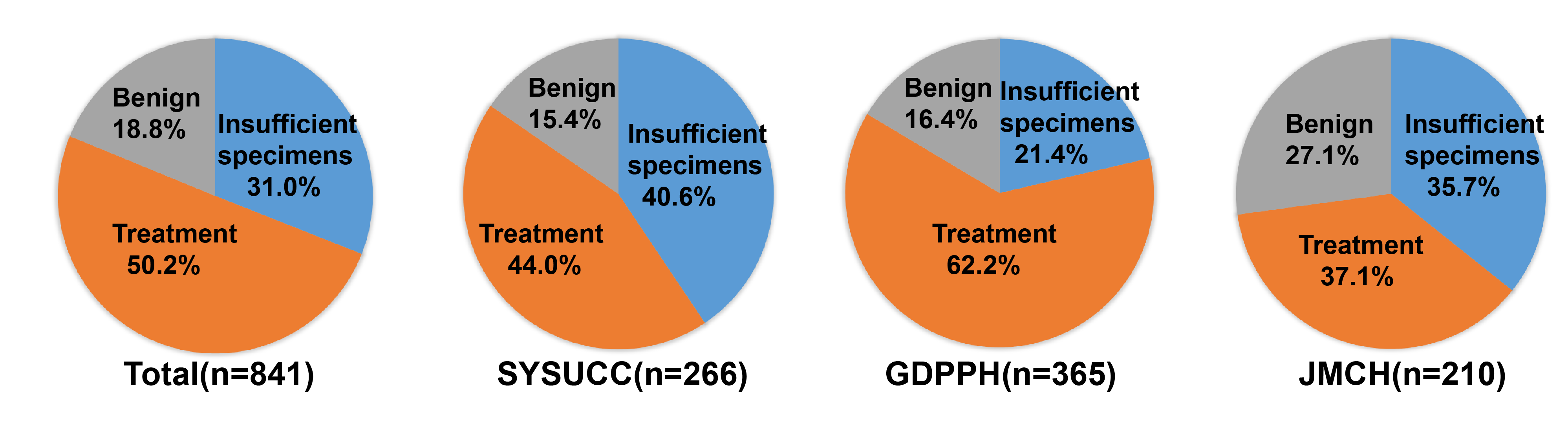

Supplement: Supplementary file 2 — Figure S2: Distribution of factors leading to rebiopsy. [file CAM4-14-e71228-s008.tif]

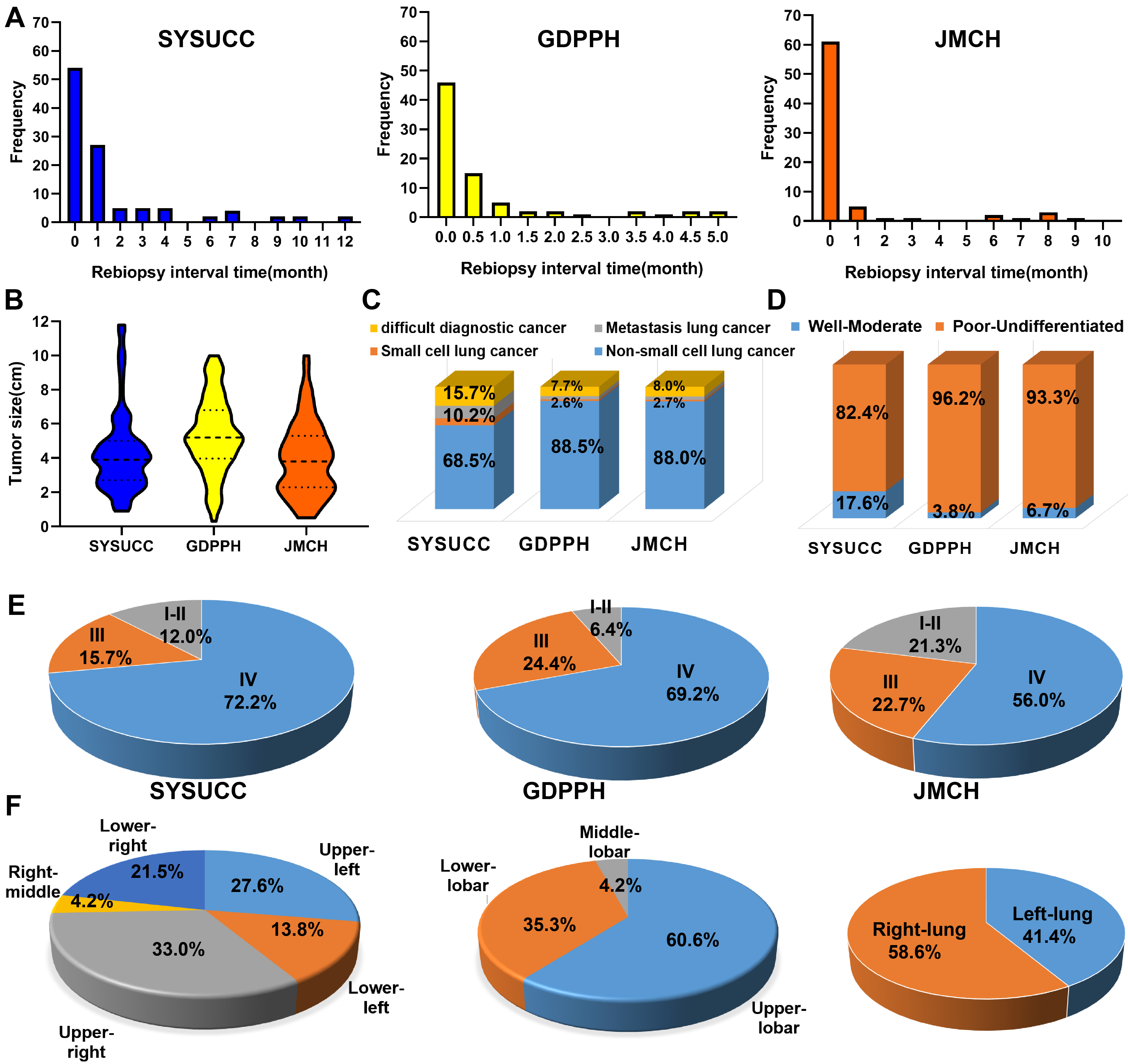

Supplement: Supplementary file 3 — Figure S3: Clinicopathological characteristics of rebiopsy patients with insufficient specimens. (A) Frequency distribution of rebiopsy interval time. (B) Distribution of tumor size. (C) Distribution of pathological histology. (D) Distribution of tumor differentiation. (E) Distribution of TNM stage. (F) Lobar site of the biopsy tissue. [file CAM4-14-e71228-s006.tif]

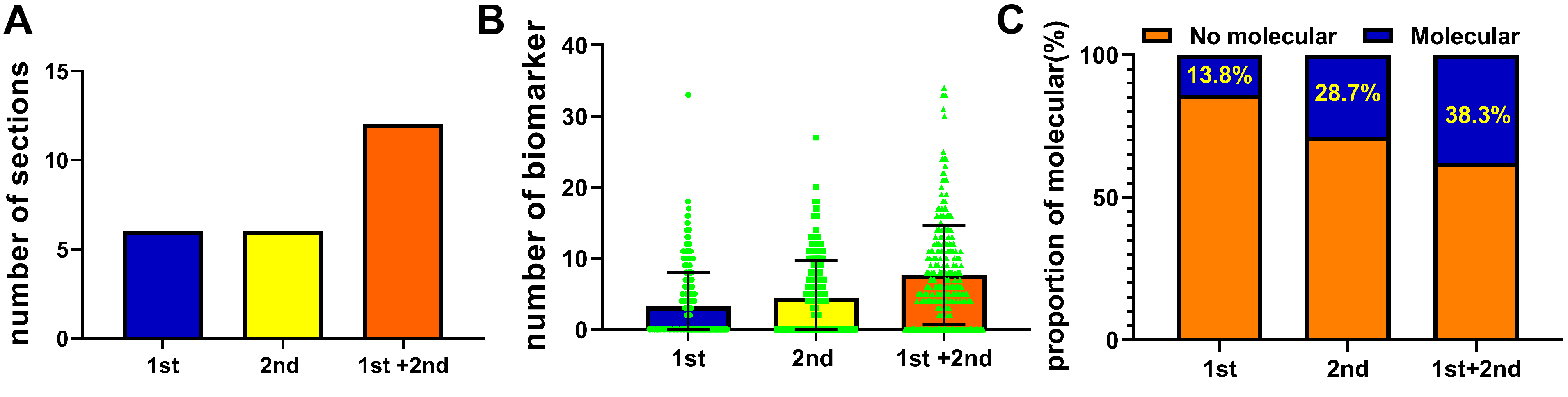

Supplement: Supplementary file 4 — Figure S4: Pathological detection needs of rebiopsy patients with insufficient specimens. (A) Number of sections with H&E staining. (B) Biomarker count for immunohistochemistry (IHC) and special staining. (C) Proportion of molecular detection. [file CAM4-14-e71228-s007.tif]

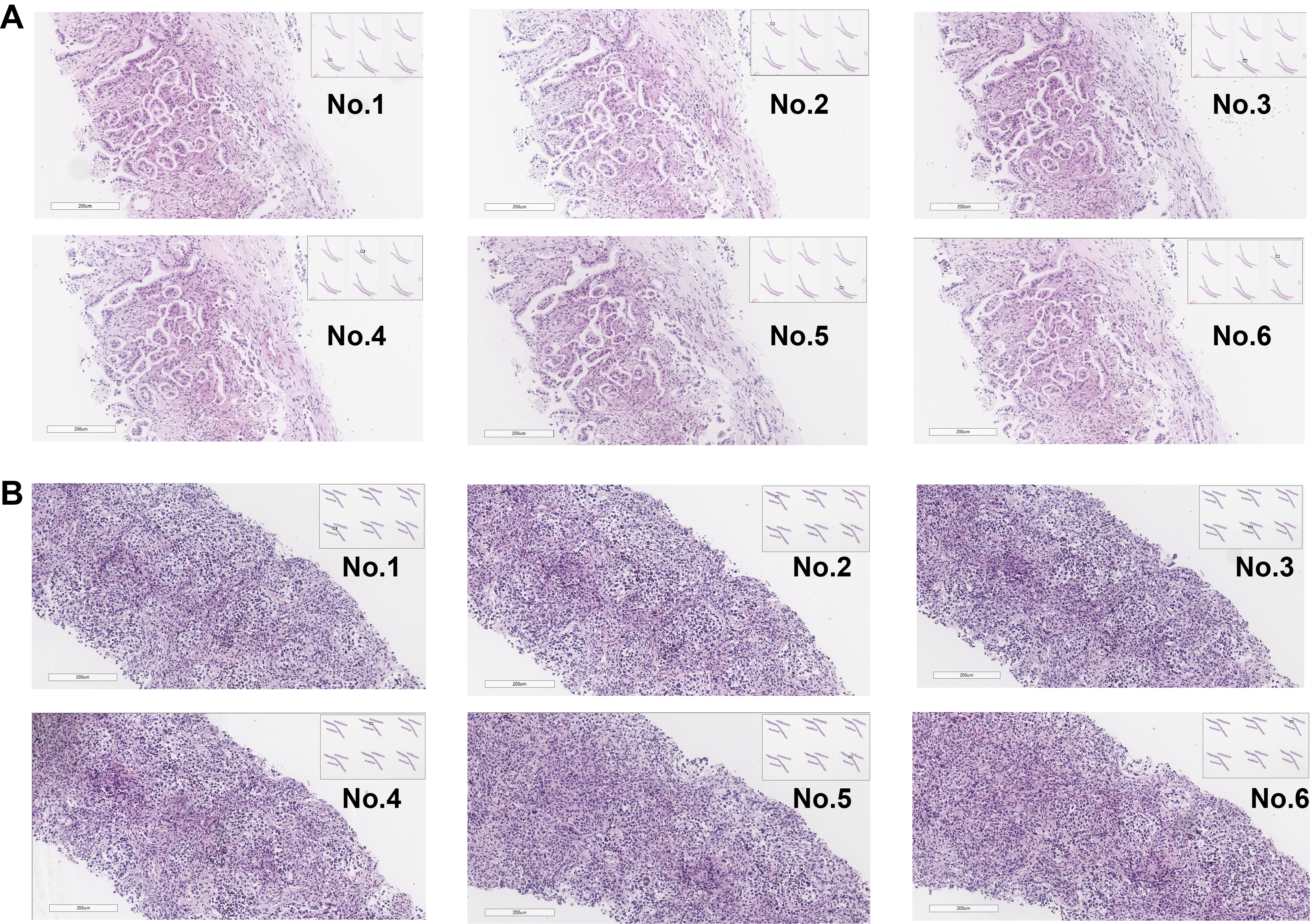

Supplement: Supplementary file 5 — Figure S5: Images of H&E staining with continuous sections for PTNBP tissues. (A) H&E images of lung adenocarcinoma. (B) H&E images of poorly differentiated carcinoma (10×). [file CAM4-14-e71228-s002.tif]
