# Supplementary material for: A Multicenter Study on Unnecessary Rebiopsies in CT‐Guided Percutaneous Transthoracic Needle Biopsy of Pulmonary Lesions
Source: Cancer Med. 2025 Sep 29;14(19):e71228. doi: 10.1002/cam4.71228 (PMC12477545; doi:10.1002/cam4.71228)
Supplement: Supplementary file 6 — Table S1: Differences in clinicopathological parameters between rebiopsy cases with insufficient specimens and those due to treatment. [file CAM4-14-e71228-s009.docx]

**Supplementary Table 1: Differences in clinicopathological parameters between rebiopsy cases with insufficient specimens and those due to Treatment**

| **Variable** | **Insufficient specimens(n=261)** | **Treatment(n=422)** | **P** |
| --- | --- | --- | --- |
| **Age(y)^a^** | 61.8±10.7 | 59.1±10.6 | **0.0014** |
| **Rebiopsy interval time(month)^a^** | 1.2±2.3 | 15.1±10.8 | **<0.0001** |
| **Gender^b^** |  |  | **0.0006** |
| Male | 177(67.8%) | 230(54.5%) |  |
| Female | 84(32.2%) | 192(45.5%) |  |
| **Histology(final)^b^** |  |  | **0.0054** |
| Non-small cell lung cancer | 209(80.1%) | 371(87.9%) |  |
| Others | 52(19.9%) | 51(12.1%) |  |
| **Differentiation^b^** |  |  |  |
| Well-Moderate | 27(10.3%) | 42(10.0%) | 0.8687 |
| Poor-Undifferentiated | 234(89.7%) | 380(90.0%) |  |
| ^a^Data are the Mean±SD,Unpaired t test | |  |  |
| ^b^Data are the Number(Proportion),Chi-square test | |  |  |
