# Supplementary material for: A Multicenter Study on Unnecessary Rebiopsies in CT‐Guided Percutaneous Transthoracic Needle Biopsy of Pulmonary Lesions
Source: Cancer Med. 2025 Sep 29;14(19):e71228. doi: 10.1002/cam4.71228 (PMC12477545; doi:10.1002/cam4.71228)
Supplement: Supplementary file 7 — Table S2: The biomarker of IHC and molecular testing. [file CAM4-14-e71228-s005.docx]

**Supplementary Table 2: The biomarker of IHC and molecular testing**

| **Biomarker** | | **Cases** | **Proportion** |
| --- | --- | --- | --- |
| **IHC** |  |  |  |
|  | TTF1 | 199 | 92.13% |
|  | p40 | 178 | 82.41% |
|  | Napsin A | 126 | 58.33% |
|  | CK5/6 | 119 | 55.09% |
|  | p63 | 95 | 43.98% |
|  | Ki‑67 | 81 | 37.50% |
| **Molecular** | |  |  |
|  | ALK | 50 | 23.15% |
|  | EGFR | 46 | 21.30% |
|  | ROS1 | 12 | 5.56% |
