# Supplementary material for: A Multicenter Study on Unnecessary Rebiopsies in CT‐Guided Percutaneous Transthoracic Needle Biopsy of Pulmonary Lesions
Source: Cancer Med. 2025 Sep 29;14(19):e71228. doi: 10.1002/cam4.71228 (PMC12477545; doi:10.1002/cam4.71228)
Supplement: Supplementary file 8 — Table S3: Relationship between clinicopathological parameters and pathological detection needs. [file CAM4-14-e71228-s004.docx]

**Supplementary Table 3 Relationship between clinicopathological parameters and pathological detection needs**

| **Variable** | **All cases** | **Biomarker number of**  **IHC and** **special staining** | | |  | **Proportion of molecular** | | |
| --- | --- | --- | --- | --- | --- | --- | --- | --- |
|  |  | **Mean** | **Difference^a^** | ***P ^b^*** |  | **Proportion** | **χ^2^** | ***P ^c^*** |
| **Gender** |  |  | -0.9837 ± 0.9246 | 0.2884 |  |  | 0.2450 | 0.6206 |
| Male | 177 | 7.972 |  |  |  | 37.3%(66/177) |  |  |
| Female | 84 | 6.988 |  |  |  | 40.5%(34/84) |  |  |
| **Age(y)** |  |  | 0.08847 ± 0.8669 | 0.9188 |  |  | 0.4094 | 0.5223 |
| ≤63 | 137 | 7.613 |  |  |  | 40.2%(55/137) |  |  |
| >63 | 124 | 7.702 |  |  |  | 36.3%(45/124) |  |  |
| **Lesion size(cm)** |  |  | 2.742 ± 0.8492 | **0.0014** |  |  | 1.409 | 0.2353 |
| ≤4.2 | 134 | 6.321 |  |  |  | 41.8%(56/134) |  |  |
| >4.2 | 127 | 9.063 |  |  |  | 34.7%(44/127) |  |  |
| **Differentiation** | |  | 4.077 ± 1.399 | **0.0039** |  |  | 0.02078 | 0.8854 |
| Well-Moderate | 27 | 4 |  |  |  | 37.0%(10/27) |  |  |
| Poor-Undifferentiated | 234 | 8.077 |  |  |  | 38.5%(90/234) |  |  |
| **Histology(final)** | |  | 5.066 ± 1.037 | **<0.0001** |  |  | 1.564 | 0.2111 |
| NSCLC | 209 | 6.646 |  |  |  | 40.2%(84/209) |  |  |
| Others | 52 | 11.71 |  |  |  | 30.8%(16/52) |  |  |
| **Lobar site** |  |  | 0.4542 ± 0.8785 | 0.6056 |  |  | 0.009615 | 0.9219 |
| Left | 108 | 7.389 |  |  |  | 38.0%(41/108) |  |  |
| Right | 153 | 7.843 |  |  |  | 38.6%(59/153) |  |  |
| **TNM Stage** |  |  | -1.328 ± 0.9146 | 0.1479 |  |  | 9.370 | **0.0022** |
| I-III | 87 | 8.540 |  |  |  | 25.3%(22/87) |  |  |
| IV | 174 | 7.213 |  |  |  | 44.8%(78/174) |  |  |
| a:Difference between means (B - A) ± SEM; b:Unpaired t test; c:Chi-square test | | | |  |  |  |  |  |
