# Supplementary material for: A Multicenter Study on Unnecessary Rebiopsies in CT‐Guided Percutaneous Transthoracic Needle Biopsy of Pulmonary Lesions
Source: Cancer Med. 2025 Sep 29;14(19):e71228. doi: 10.1002/cam4.71228 (PMC12477545; doi:10.1002/cam4.71228)
Supplement: Supplementary file 9 — Table S4: Multivariate analyses of clinicopathological parameters for pathological detection needs (n = 261). [file CAM4-14-e71228-s001.docx]

**Supplementary Table 4 Multivariate analyses of clinicopathological parameters for pathological detection needs (n=261)**

| **Variables** | **IRR^a^** | **95% CI^b^** | ***P* value^c^** |
| --- | --- | --- | --- |
| **Gender** (male vs. female) | 1.146 | 0.909-1.444 | 0.248 |
| **Age** (>63years vs. ≤63) | 1.019 | 0.832-1.249 | 0.855 |
| **Tumor size** (>4.2 cm vs. ≤4.2) | 1.277 | 1.038-1.571 | **0.021** |
| **Differentiation** (low vs. high ) | 1.606 | 0.909-2.839 | 0.103 |
| **Histology** (NSCLC vs. others) | 0.6 | 0.471-0.764 | **<0.001** |
| **Lobar site** (right vs. left) | 1.02 | 0.824-1.262 | 0.859 |
| **TNM** (IV vs. I-III) | 0.853 | 0.693-1.05 | 0.133 |
| a: incidence-rate ratios b: confidence interval c: poisson regression | | | |
