# Supplementary material for: A Multicenter Study on Unnecessary Rebiopsies in CT‐Guided Percutaneous Transthoracic Needle Biopsy of Pulmonary Lesions
Source: Cancer Med. 2025 Sep 29;14(19):e71228. doi: 10.1002/cam4.71228 (PMC12477545; doi:10.1002/cam4.71228)
Supplement: Supplementary file 10 — Table S5: Clinicopathological parameters of patients stained H&E with continuous sections. [file CAM4-14-e71228-s003.docx]

**Supplementary Table 5 Clinicopathological parameters of patients stained H&E with continuous sections**

| **Variable** | **All cases**  **(n=1079)** | **Retrospective cases**  **(n=532)** | **Prospective cases**  **(n=547)** |
| --- | --- | --- | --- |
| **Gender** |  |  |  |
| Male | 690(63.9%) | 344(64.7%) | 346(63.3%) |
| Female | 389(36.1%) | 188(35.3%) | 201(36.7%) |
| **Age(y)^*^** | 59.5/60.0 | 59.0/60.0 | 59.9/61.0 |
| **Histology(final)** |  |  |  |
| Non-small cell lung cancer | 580(53.8%) | 271(50.9%) | 309(56.5%) |
| Small cell lung cancer | 26(2.4%) | 11(2.1%) | 15(2.7%) |
| Metastasis lung cancer | 117(10.8%) | 48(9.0%) | 69(12.6%) |
| Difficult diagnostic cancer | 110(10.2%) | 53(10.0%) | 57(10.4%) |
| Benign | 246(22.8%) | 149(28.0%) | 97(17.7%) |
